# Curricular changes and interim posts during Covid-19: graduates’ perspectives

**DOI:** 10.1186/s12909-022-03477-6

**Published:** 2022-05-31

**Authors:** Mary Goble, Andrew Whitfield, Joseph Ogden-Newton, Pirashanthie Vivekananda-Schmidt

**Affiliations:** 1 The Medical School, University of Sheffield, Sheffield, UK; 2 Brighton and Sussex Medical School, Brighton, UK

**Keywords:** Communication, Curriculum, Legitimacy, Preparedness, Programmatic assessment, Covid-19

## Abstract

**Background:**

During the COVID-19 pandemic UK medical schools facilitated the early graduation of their final-year medical students to ‘Foundation interim Year 1 (FiY1) doctors’ through amendments made to curricula and final assessment. Such changes gave opportunity for evaluation. This study therefore aimed to explore 1) graduate perspective on the implementation of FiY1 and 2) how changes to course structures have affected self-reported preparedness for work.

**Methods:**

Questionnaire surveys using Likert scale and free-text responses (*n* = 45), and semi-structured interviews (*n* = 7) were conducted with FiY1s from two UK medical schools contrasting in the amendments made to course structures. Data were analysed using quantitative methods and thematic analysis; 44% (*n* = 20) of respondents believed that governing health bodies had not communicated sufficiently prior to starting work.

**Results:**

Graduates who had sat modified practical and written examinations reported ‘legitimacy’ and feeling more prepared compared to having not sat examinations (practical 100%, *n* = 17; written 88.3%, *n* = 15). Graduates from both schools agreed that carrying out assistantships as originally scheduled would have made them feel more prepared (91.1%, *n* = 41).

**Conclusions:**

The implementation of FiY1 was largely well received by graduates yet assistantship programmes may fulfil a similar role in normal times. Medical schools and governing bodies must ensure effective communication channels exist with students in order to better prepare them for their first posts, especially in times of crisis. Additionally, final examinations contribute to feelings of preparedness for work and instil a sense of legitimacy, a finding which is relevant to working within the current programmatic assessment structure.

**Supplementary Information:**

The online version contains supplementary material available at 10.1186/s12909-022-03477-6.

## Background

Due to the extraordinary nature of the Covid-19 pandemic, a joint statement was released by UK governing bodies in March 2020 encouraging the accelerated graduation of all final year medical students [1, 2]. In order to comply, medical schools implemented changes to learning and assessment and facilitated the early provisional registration of their final year students with the General Medical Council (GMC), allowing them to start work as Foundation “interim” Year 1 (FiY1) doctors. This post was to be carried out prior to commencing the usual two-year Foundation programme that UK graduates complete on graduation.

In a time when novel approaches to education, such as programmatic assessment (utilising a multitude of data points for assessment, optimised for learning) and longitudinal integrated clerkships (meeting clinical competencies using simultaneous and integrated strands of teaching) are under investigation [3–6], the omission of current curricula elements due to the pandemic allows for an opportunity to understand their influence on students. In addition, there has been little evaluation on the implementation of the ‘interim’ post from a graduate perspective. We surveyed and interviewed graduates from two UK medical schools in the weeks immediately following commencement of their FiY1 posts collecting opinions on: levels of communication received prior to starting work; how modifications to curricula have contributed to self-reported preparedness for work; and how graduates believe they could have been better supported during the process.

### Preparedness

Preparedness for Foundation training is a statistic collected annually via the National Training Survey using a six-point Likert scale to evaluate the statement ‘I was adequately prepared for my first foundation post’. However, during the pandemic, the 2020 survey was modified in order to better capture trainee wellbeing, workload, burnout and patient safety [7]. This shift in focus has meant the preparedness of new graduates was not assessed, giving further impetus to our study.

### Amendments to curricula

The two medical schools studied were chosen due to their comparable course structures yet differing strategies when it came to alteration of final assessments. Students from both medical schools were due to undergo written and OSCE (Objective Structured Clinical Examination) practical examinations in March 2020. Medical School A cancelled both forms of examination and instead confirmed competencies based on previous attainments and in-placement assessments. Students from Medical School B undertook modified online written examinations and an abbreviated OSCE earlier than was originally planned. Usually, Medical School A would have carried out a six-week ‘assistantship’ shadowing F1 doctors, and School B, a ‘preparation-for-practice’ module teaching procedural tasks such as ‘To Take Out’ (TTO) medication prescription, composing discharge summaries and writing drug charts. Both of these curricula items were cancelled.

### Information received

Prior to starting work, duties and professional behaviours expected during FiY1 as well as recommendations for supervision and induction were outlined by the GMC, British Medical Association (BMA) and UK foundation programme (UKFPO) via email correspondence [8]. Recommendations included remuneration, indemnity, access to online training resources, providing a named clinical supervisor and implementing a ‘buddy’ system with current foundation trainees. During FiY1, graduates had responsibility for taking notes, ordering investigations, performing bedside clinical skills, completing discharge summaries and prescribing medication, the latter only being the case if they had successfully passed the Prescribing Safety Assessment exam. Health Education England (HEE) facilitated a webinar to understand and remedy concerns raised by graduates. Updates on FiY1 allocation were sent to graduates via email by the course administrators of both medical schools. These were followed by provision of various online educational resources relevant for foundation training. In addition, wellbeing sessions were provided by foundation schools both prior to and during FiY1 posts. FiY1s from Medical School A and B were deployed from May 2020 until starting their usual Foundation Year 1 post in August 2020, all students had the opportunity to apply for a post.

### Aims and goals

The aim of this study was to explore how changes to course structures made as a result of the COVID-19 influenced graduate opinions related to starting work.

The specific goals of the study were to answer the following research questions:

1. How did graduates perceive the implementation of the FiY1 post?
2. How have changes to course structures affected graduates’ feelings of preparedness for their first posts as doctors?

## Method

### Participants

Only FiY1 doctors having graduated Summer 2020 from two UK medical schools, A and B, were eligible to participate in this study. Invitations to take part were distributed via email, in closed social media groups, and on medical school virtual notice boards although participation was not mandatory. Two hundred forty-five graduates were eligible from Medical School A and 130 from B. Participants were provided with information regarding the details of the study (Additional file 1), were screened for eligibility only allowing the user to proceed if answering ‘yes’ to currently working as an FiY1 and being from either Medical School A or B, and were required to give informed virtual written consent that their responses could be used for publication.

### Ethics

Ethical Approval for this study was obtained from Sheffield Medical School Research Ethics Committee, 034055 approved 27/04/2020.

### Questionnaire

A novel questionnaire was devised using feedback from a focus group of three graduates who were then deemed unable to take further part in the study. Questionnaires were distributed by medical school administrators via email to graduates. Participants could complete the final questionnaire (Additional file 2) following an eligibility screen confirming current employment as an FiY1. The questionnaire consisted of 22 questions, with two additional questions for graduates of Medical School B regarding the alternative examinations they had undertaken. Questions investigated respondent agreement with statements using 6-point Likert scales (Strongly agree, Agree, Neither agree nor disagree, Disagree, Strongly disagree, Don’t know), yes or no questions, or free-text responses. Questions explored communication, assessment, assistantship, preparedness and well-being, themes derived by the researchers following focus groups. While the psychometric properties of the questionnaires were not formally assessed, construct validity was improved by use of a relevant focus group for its construction. Structural validity was also preserved using Likert scales as per the National Training Survey [7].

Participants were asked to enter an email address at the end of the questionnaire if they consented to be contacted for an interview. This email address was separated from survey responses when data were downloaded for analysis. Data were therefore anonymous and devoid of personal information. Questionnaire responses were collected 1st June-3rd July 2020.

### Interview

Interviews were carried out and recorded via GoogleHangouts [9] and took place from 22nd June-3rd July 2020. Interviews were semi-structured with five scripted questions informed by the questionnaire data collated by the researchers. Interview script and prompts is available as Additional file 3. Interviews were approximately 30 minutes in length and carried out by one researcher. No incentives were provided for interview owing to lack of study funding. A mix of both quantitative and qualitative study methods allowed for triangulation of data and broader conclusion to be drawn.

### Analysis

Data were analysed in SPSS V26.0 [10]. Demographics were compared using either t-tests or *X*^*2*^ as appropriate. While kurtosis values of Likert scale data largely fell within acceptable values of − 1.96 to + 1.96 for both medical schools, all data were shown to be non-normal by significant Shapiro-Wilk values. Non-parametric tests were therefore performed. Responses from schools A versus B for Likert-scale questions were compared using Mann-Whitney U tests. False discovery rate (FDR) corrections were applied to account for multiple comparisons with q = 0.05 in order to reduce the rate of type I errors.

Thematic analysis was performed on the qualitative data from free text answers and interview transcripts using the method outlined by Kiger and Varpio [11]. Initial themes were determined by at least two researchers independently with common themes then agreed upon. Percentages outlined in free text and interview answers reflect the proportion of respondents whose answers fell within a given theme. Themes and number of responses falling into each category are outlined in Additional files 4 and 5.

## Results

A response rate of 11.4% (*n* = 28) was achieved for Medical School A and 13.1% (*n* = 17) for Medical School B. Following FDR correction of Likert-scale responses, no significant differences were found between answers from Schools A and B. Additionally there were no significant differences in terms of demographics for age (A: *M* = 24.07 *SD* = 1.39 B: *M* = 24.41 *SD* = 1.50) *t* (45) = 0.7739, *p* = .4432 and in terms of gender *X*^*2*^ (1, *N* = 45) = 1.1817, *p* = .277006. Quantitative data from Likert-scale questions from both schools were therefore combined for each question. Qualitative data from free text questions and interviews were separated by medical school to allow for opinions on differences made to course structures to be articulated.

### Likert scale responses (Fig.1)

Generally, FiY1s agreed that their medical schools had communicated sufficiently to put them at ease during the current COVID-19 crisis (71.1% *n* = 32). However, a large proportion of respondents (45% *n* = 20) believed that governing bodies (GMC, HEE, Medical Schools Council) had not communicated sufficiently. The majority of respondents agreed that they had felt prepared when starting FiY1 (68.9% *n* = 31). However, no respondents indicated the ‘strongly agree’ option for this question (Fig. 2).

**Fig. 1:**
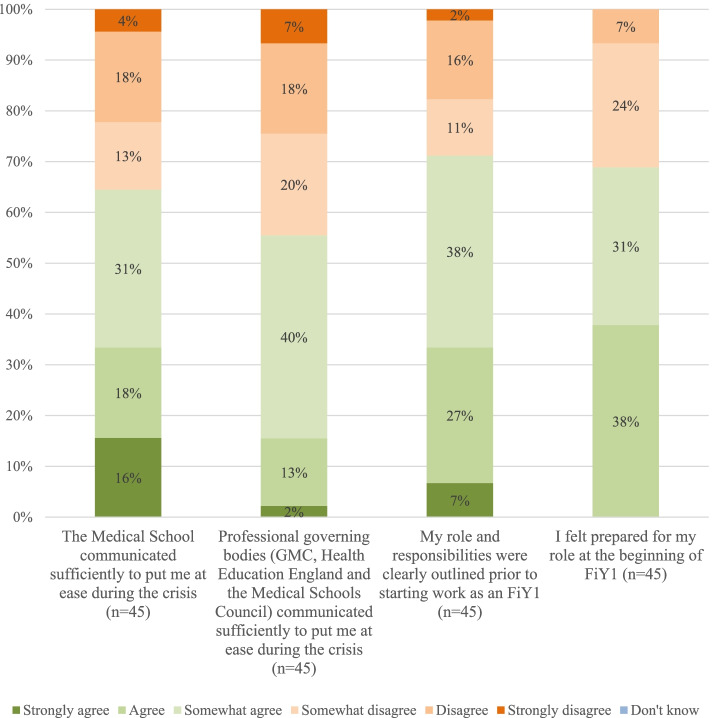
Communication-related items (cumulative frequencies for Likert-scale responses)

**Fig. 2:**
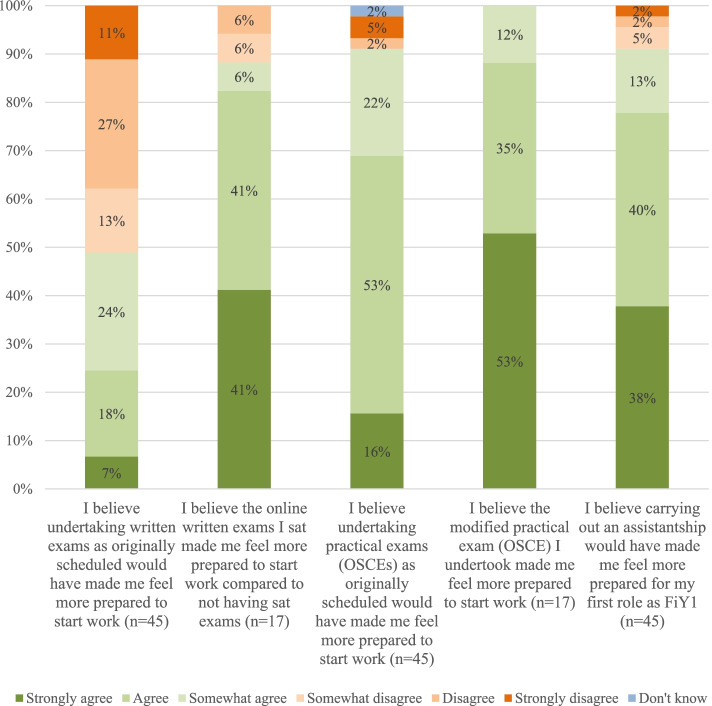
Assessment-related items (cumulative frequencies for Likert-scale responses)

The majority of graduates agreed (91.1% *n* = 41) that undertaking practical OSCE exams as originally scheduled would have made them feel more prepared and all graduates of medical school B (100% *n* = 17) who sat a modified OSCE reported that this increased their feelings of preparedness. Graduates also generally agreed that modified online written exams helped in their preparedness for work compared to not sitting exams (88% *n* = 15). Respondents agreed that assistantships programmes undertaken as normal would have made them feel more prepared (91% *n* = 41).

### Free text and interview responses

Eight participants consented to be contacted for interview, one of whom was lost to follow-up. Four graduates were interviewed from Medical School A and three from B. Interviewees from both schools reported wishing they had been kept “more in the loop”; as there had been periods of “radio-silence” and “fractured” communication at times. School A graduates reported that information was usually only available through their student ‘Year Representative’ and wished for more direct communication from the medical school. One student felt that they were “nobody’s direct responsibility” since graduating as it was not clear who had the ultimate responsibility for relaying information.

In terms of examinations undertaken, one graduate from Medical School A reported their knowledge as “not feeling fresh” while in comparison a graduate of Medical School B reported feelings of “legitimacy” having succeeded in passing their modified exams and having “confidence when interacting with patients” because of this. Another interviewee from Medical School B reported “[I] don’t think having done exams ‘actually’ prepared me more, it was more about ‘feeling’ more prepared (sic)”.

Graduates of both universities reported finding that “exam knowledge doesn’t help with day-to-day (as an FiY1)” and that exams would “have made minimal difference to the difficulty of starting a new job”. Many interviewees wished for more formal teaching on procedural tasks such as “TTOs or discharge summaries”.

Graduates of Medical School A were least confident in managing acutely unwell patients and making decisions independently (32.1% *n* = 9, 17.9% *n* = 5 respectively) and School B in prescribing and working outside of their competencies due to unclear responsibilities (41.2% *n* = 7, 17.6% *n* = 3 respectively). Both sets of graduates felt most confident with their clinical skills (School A 34.5% *n* = 6, School B 33.3% *n* = 5). Most students accessed no resources at all to support their well-being and mental health (77.8% *n* = 35), 50% (*n* = 5) of those who did used mindfulness and yoga practices. More than half (58.8% *n* = 10) of those who accessed additional academic resources did so by watching online lectures and courses from private enterprises. In terms of additional support, Medical School B graduates (46.2% *n* = 6) would have liked to have completed their ‘prep-for-practice’ module as usual.

## Discussion

In relation to research question one, many FiY1s would have liked more direct communication from their medical schools and especially from governing bodies with a large proportion believing that communication from governing bodies was insufficient (45% *n* = 20). Despite this, most students felt their roles and responsibilities were made clear prior to starting work (71.1% *n* = 32) and most respondents felt prepared for their FiY1 post (68.9% *n* = 31). It was however noted that no students chose the ‘strongly agree’ option when describing their preparedness to start work highlighting the fact that every junior doctor felt in some way that more could have been done. Communication with students can be optimized through timely distribution of surveys, responding to common concerns in weekly emails, and providing regular opportunities to meet with medical school faculty members as in one American medical school model initiated during the pandemic [12]. Using such methods to maintain communication channels with graduates would have alleviated concerns of information not being relayed promptly.

In relation to research question two, graduates perceived that the academic content tested in final year exams was less useful for work than knowledge of procedural tasks, however, passing finals, even in a modified format, gave them a feeling of “legitimacy” and “confidence”. This was true for both written and practical examinations. As many medical schools increase their focus on programmatic assessment through utilization of placement-based assessment and small group tutor feedback, final examinations are considered to be of relative not absolute importance [13, 14]. However, we suggest that final examinations continue to play an important role in providing external validation for new doctors, which should be taken into consideration when planning future curricular revisions.

Graduates reported practical examinations to contribute more to their feelings of preparedness than written examinations with 100% (*n* = 17) of those who undertook modified OSCE examinations agreeing that these contributed to feelings of preparedness for work. This is classically illustrated by Miller’s ‘Framework for clinical assessment’ [15] which favours ‘shows how’ to ‘knows how’ as a mode of testing. OSCEs are also often considered to be superior to written examinations in terms of their range of assessment and versatility [16–18]. We suggest that not only are OSCEs valuable in terms of their quality of assessment but also in terms of student preference.

A large proportion of graduates who felt the need to supplement their learning did so by watching online webinars organized by private enterprises (58.8% *n* = 10). Whilst these organizations are usually led by doctors and provide reliable information, medical schools and governing bodies have the ultimate responsibility for preparing their students for work.

Teaching focused on procedural tasks such as TTOs and discharge summaries was found to be lacking by graduates. The ‘buddy’ system, initially recommended by the UKFPO, was not discussed by graduates and has elsewhere been reported as difficult to implement [8, 19]. Proper organisation of such a scheme may have aided in learning. However, these are skills often gained through shadowing on ‘assistantship’ programmes; a part of curricula graduates missed out on, and one respondents largely agreed would have made them feel more prepared for work (91.1% *n* = 41). Assistantships are known to improve self-reported preparedness for work and in lieu of these, sufficient resources should have been provided to supplement learning [20–22].

At no point did graduates report anything to suggest the superiority of FiY1 over assistantship programmes. This point has been argued by Butt and Umaskanth [23] who feel that the extra responsibility of an FiY1 role better prepares doctors for work and recommend that the FiY1 programme becomes the norm after final examinations. More focussed studies are necessary to sufficiently answer this question. It is however interesting to note that FiY1, providing all of the responsibilities of a doctor, with maximal supervision, may fulfil Miller’s fabled ‘Does’ rung on the pyramid of clinical competence [15, 23]. By integrating robust feedback systems to the role, FiY1 may yet prove useful as a fine-tuning tool for graduates before independent clinical practice begins.

This study was limited to two UK medical schools. As such, small sample sizes and low response rates meant variation across schools was difficult to detect and recommendations specific to teaching styles and aspects of curricula were therefore harder to make. Incentives for completion of questionnaires and interview could have been provided if funding were available and may have improved response rates. Other limitations lay in the external factor of the COVID-19 pandemic. One survey of final year medical students found that the pandemic itself had affected students’ perceived preparedness for foundation training, independent of changes made to curricula [24]. Due to these multiple confounders, it was decided best not to directly compare our results to the National Training Survey from years past for example. Additionally, self-reported preparedness as a measure does not necessarily correlate with actual preparedness and so should not be mistaken for competence [25, 26]. However, self-reported preparedness can provide an opportunity for reflection and self-assessment, important attributes for a doctor [27]. Furthermore, medical schools and governing bodies should take an interest in the wellbeing of their new doctors and see this measure as a way to gauge the mindsets of graduates entering their first posts.

## Conclusion

Lessons we are able to learn from the introduction of the FiY1 programme include the importance of maintaining effective communication channels with students, especially in times of crisis. Having said this, the roll-out of the FiY1 programme was largely seen as positive by graduates. The majority of respondents felt there to be sufficient communication from their medical school and from governing bodies, that their roles and responsibilities were clear and that they were prepared for the post.

The role of assistantships in preparing students for work is supported by this study as many skills that graduates felt they were lacking would have been consolidated in normal times with such programmes. More focussed studies are necessary to determine whether FiY1 shows any superiority over assistantship programmes.

Furthermore, final examinations, especially OSCEs, contribute to feelings of preparedness. Finals were found to instil feelings of ‘legitimacy’ and ‘confidence’. The external validation gained by such exams should be taken into account while medical schools shift towards programmatic assessment frameworks.

## Supplementary Information

**Additional file 1.** Participation information and consent form. Information provided to eligible participants with consent sought before proceeding.

**Additional file 2.** Questionnaire. Initial questionnaire distributed to participants.

**Additional file 3.** Semi-structures interview questions. Questions and prompts asked in semi-structured interview. Questions informed by questionnaire responses.

**Additional file 4.** Themes from thematic analysis of free text responses. Derived themes from thematic analysis of free text responses.

**Additional file 5.** Themes from thematic analysis of interview responses. Derived themes from thematic analysis of interview responses.

## Data Availability

Data related to the study can be relayed by the corresponding author.

## Abbreviations

FiY1: Foundation interim Year 1
GMC: General Medical Council
OSCE: Objective Structured Clinical Exam
TTO: To Take Out
BMA: British Medical Association
UKFPO: United Kingdom Foundation Programme Office
HEE: Health Education England
FDR: False Discovery Rate
