# Supplementary material for: Curricular changes and interim posts during Covid-19: graduates’ perspectives
Source: BMC Med Educ. 2022 May 31;22:413. doi: 10.1186/s12909-022-03477-6 (PMC9152820; doi:10.1186/s12909-022-03477-6)
Supplement: Supplementary file 1 — Additional file 1. Participation information and consent form. Information provided to eligible participants with consent sought before proceeding. [file 12909_2022_3477_MOESM1_ESM.docx]

**Appendix 1: Participation information and consent form**

Supporting medical students in times of crisis: Lessons from the COVID-19 interim Foundation post

Important: interim Foundation Year 1 (FiY1) doctors are Foundation doctors who have been granted provisional registration by the GMC earlier than usual, as part of the UK Government's response to the Covid-19 pandemic.

You are being invited to take part in this study surveying FiY1s. It is important that you understand why this work is being carried out and what it will involve. Please take the time to read the participant information sheet at: https://tinyurl.com/ycc4koto. If you have any questions, please do not hesitate to contact the researchers (contact details can be found on the participant information sheet and at the end of the survey).

Please read each of the statements below before proceeding.

- I have read and understood the project information sheet. If your answer to this question is NO, please do not proceed with this consent form until you are fully aware of what your participation in the project will mean.

- I have been given the opportunity to ask questions about the project.

- I understand that taking part in the project will include completing a questionnaire with the option of answering questions as part of a semi-structured interview.

- I understand that my taking part is voluntary.

- I understand that no personal data will be recorded during this study, unless I choose to partake in interviews. In this case, it is at my discretion to provide an email address which will be separated from the rest of the data and destroyed at the outcome of interviews. There are no negative consequences should you not wish to partake in interviews.

- I understand and agree that my words may be quoted in publications, reports, web pages, and other research outputs. This data will be anonymous, and participants will not be identifiable.

By taking part I agree to give consent.
