# Supplementary material for: Curricular changes and interim posts during Covid-19: graduates’ perspectives
Source: BMC Med Educ. 2022 May 31;22:413. doi: 10.1186/s12909-022-03477-6 (PMC9152820; doi:10.1186/s12909-022-03477-6)
Supplement: Supplementary file 2 — Additional file 2. Questionnaire. Initial questionnaire distributed to participants. [file 12909_2022_3477_MOESM2_ESM.docx]

**Appendix 2: Questionnaire**

| **Demographics** | | | | | | |
| --- | --- | --- | --- | --- | --- | --- |
| I am currently working as an interim F1 | Yes | | | No | | |
| Age |  | | | | | |
| Gender |  | | | | | |
| Which medical school did you graduate from? |  | | | | | |
| **Communication** | | | | | | |
| The Medical School communicated sufficiently to put me at ease during the crisis. | *Strongly agree* | *Agree* | *Neither agree nor disagree* | *Disagree* | *Strongly disagree* | *Don’t know* |
| Professional governing bodies (GMC, Health Education England and the Medical Schools Council) communicated sufficiently to put me at ease during the crisis. | *Strongly agree* | *Agree* | *Neither agree nor disagree* | *Disagree* | *Strongly disagree* | *Don’t know* |
| My role and responsibilities were clearly outlined prior to starting work as an FiY1. | *Strongly agree* | *Agree* | *Neither agree nor disagree* | *Disagree* | *Strongly disagree* | *Don’t know* |
| **Preparedness** | | | | | | |
| I felt prepared for my role at the beginning of FiY1. | *Strongly agree* | *Agree* | *Neither agree nor disagree* | *Disagree* | *Strongly disagree* | *Don’t know* |
| **Assessment** | | | | | | |
| I believe undertaking written exams as originally scheduled would have made me feel more prepared to start work. | *Strongly agree* | *Agree* | *Neither agree nor disagree* | *Disagree* | *Strongly disagree* | *Don’t know* |
| Medical School B Only  I believe the online written exams I sat made me feel more prepared to start work compared to not having sat exams. | *Strongly agree* | *Agree* | *Neither agree nor disagree* | *Disagree* | *Strongly disagree* | *Don’t know* |
| I believe undertaking practical exams (OSCEs) as originally scheduled would have made me feel more prepared to start work. | *Strongly agree* | *Agree* | *Neither agree nor disagree* | *Disagree* | *Strongly disagree* | *Don’t know* |
| Medical School B Only  I believe the modified practical exam (OSCE) I undertook made me feel more prepared to start work. | *Strongly agree* | *Agree* | *Neither agree nor disagree* | *Disagree* | *Strongly disagree* | *Don’t know* |
| I believe carrying out an assistantship would have made me feel more prepared for my first role as FiY1. | *Strongly agree* | *Agree* | *Neither agree nor disagree* | *Disagree* | *Strongly disagree* | *Don’t know* |
| **Confidence** | | | | | | |
| What were you most worried about starting FiY1? |  | | | | | |
| What do you feel most confident in dealing with in FiY1? |  | | | | | |
| What do you feel least confident in dealing with in FiY1? |  | | | | | |
| **Wellbeing** | | | | | | |
| Did you access any resources or support for well-being and mental health to prepare yourself for FiY1? | *Yes* | | | *No* | | |
| If yes, please describe below and explain whether they were useful. |  | | | | | |
| Did you use any additional resources, beyond what was provided by the medical school, for academic support in preparing for FiY1? | *Yes* | | | *No* | | |
| If yes, please describe below and explain whether they were useful. |  | | | | | |
| What type of additional support would you have liked to have had access to? |  | | | | | |
| I have been adequately supported during my FiY1 role so far. | *Strongly agree* | *Agree* | *Neither agree nor disagree* | *Disagree* | *Strongly disagree* | *Don’t know* |
| An “F3” career break is something I am considering taking following completion of my foundation training. | *Strongly agree* | *Agree* | *Neither agree nor disagree* | *Disagree* | *Strongly disagree* | *Don’t know* |
| I am more likely to take an “F3” career break as a result of the current COVID-19 crisis. | *Strongly agree* | *Agree* | *Neither agree nor disagree* | *Disagree* | *Strongly disagree* | *Don’t know* |
| Please highlight any concerns you may have regarding COVID-19. |  | | | | | |
| If you are happy to be contacted for a short virtual interview to further explore your views, please provide an email address below. Interviews will be conducted via Google Hangout; you will be contacted to arrange a date and time which suits you. Email addresses will be detached from the data prior to analysis. | | | | | | |
