# Supplementary material for: Curricular changes and interim posts during Covid-19: graduates’ perspectives
Source: BMC Med Educ. 2022 May 31;22:413. doi: 10.1186/s12909-022-03477-6 (PMC9152820; doi:10.1186/s12909-022-03477-6)
Supplement: Supplementary file 3 — Additional file 3. Semi-structures interview questions. Questions and prompts asked in semi-structured interview. Questions informed by questionnaire responses. [file 12909_2022_3477_MOESM3_ESM.docx]

**Appendix 3: Semi-structures interview questions**

| How did you feel about the amount of information from medical school/ governing bodies before starting FiY1? What more information would you have liked? | |
| --- | --- |
| Medical school A: Do you think sitting online exams like other medical schools would have made you feel more prepared? Why? | Medical school B: The vast majority of people felt like sitting online exams made them feel more prepared. Why do you think this is?  How did you think the modified written exam prepared you?  How do you think the modified OSCE prepared you?  How did it differ from sitting a full exam? |
| In practice now, are you comfortable in your role, doing the job you are assigned to do? How could you feel more supported? Prompt night shifts and dealing with patients alone if not brought up. How was your induction? | |
| During medical school how well did the teaching prepare you for the day-to-day tasks you’re expected to do as an F1? Prompt procedural things like TTOs and IT if not brought up | |
| Overall, how could the medical school have done better to help you feel more prepared? | |
| Finally, we’ve found that lots of people expect to feel burnout and/or take a break after foundation is finished, do you and why? | |
