# Supplementary material for: Curricular changes and interim posts during Covid-19: graduates’ perspectives
Source: BMC Med Educ. 2022 May 31;22:413. doi: 10.1186/s12909-022-03477-6 (PMC9152820; doi:10.1186/s12909-022-03477-6)
Supplement: Supplementary file 4 — Additional file 4. Themes from thematic analysis of free text responses. Derived themes from thematic analysis of free text responses. [file 12909_2022_3477_MOESM4_ESM.docx]

**Appendix 4: Themes from thematic analysis of free text responses**

|  | | **Responses which fell into theme** | |
| --- | --- | --- | --- |
| **Question** | **Theme** | **Medical school A** | **Medical school B** |
| What were you most worried about starting FiY1? | Lack of support | 6 | 2 |
|  | Acutely unwell patients | 8 | 0 |
|  | Working independently | 4 | 0 |
|  | Insufficient medical knowledge | 5 | 1 |
|  | Software/admin concerns | 3 | 2 |
|  | Prescribing | 5 | 3 |
|  | Unclear roles and responsibilities | 0 | 3 |
|  | Unfair contract | 0 | 1 |
|  | Catching COVID | 0 | 1 |
| What do you feel most confident in dealing with in FiY1? | Clinical skills | 10 | 5 |
|  | Prescribing | 3 | 0 |
|  | Ward work | 8 | 7 |
|  | Clerking | 5 | 0 |
|  | Communicating with patients | 3 | 0 |
| What do you feel least confident in dealing with in FiY1? | Making decisions independently | 5 | 5 |
|  | Managing acutely unwell patients | 9 | 0 |
|  | Prescribing | 4 | 7 |
|  | Referring patients | 2 | 1 |
|  | IT and administration | 4 | 2 |
|  | Clinical skills | 1 | 2 |
