# Supplementary material for: Curricular changes and interim posts during Covid-19: graduates’ perspectives
Source: BMC Med Educ. 2022 May 31;22:413. doi: 10.1186/s12909-022-03477-6 (PMC9152820; doi:10.1186/s12909-022-03477-6)
Supplement: Supplementary file 5 — Additional file 5. Themes from thematic analysis of interview responses. Derived themes from thematic analysis of interview responses. [file 12909_2022_3477_MOESM5_ESM.docx]

**Appendix 5: Themes from thematic analysis of interview responses**

|  | **Responses which fell into theme** | |
| --- | --- | --- |
| **Theme** | **Medical school A** | **Medical school B** |
| Concerns related to general preparedness for FiY1 | 6 | 10 |
| Criticisms of communication received related to job | 15 | 22 |
| Clarity of expected duties of the job | 3 | 7 |
| Anticipated concerns surrounding decision making as a new doctor | 4 | 1 |
| Concerns of induction received | 6 | 10 |
| Concerns related to staffing | 1 | 0 |
| Expected routes for escalation to seniors | 5 | 1 |
| Concerns as a result of changes to exams/curricula | 7 | 25 |
| Specific concerns related to discharge letters | 4 | 4 |
| Pastoral support received and wished for | 4 | 19 |
| Wishes for practical skill teaching | 8 | 3 |
| Plans after foundation training | 2 | 3 |
| Additional resources offered and wished for | 5 | 0 |
| Previous placement experience | 2 | 0 |
| Night shifts expectations | 2 | 4 |
| COVID-19 specific concerns | 4 | 2 |
| Medical school specific items | 0 | 2 |
